# Robotic spleen-preserving laparoscopic distal pancreatectomy: a single-centered Chinese experience

**DOI:** 10.1186/s12957-015-0671-x

**Published:** 2015-09-17

**Authors:** Yang Liu, Wen-Bin Ji, Hong-Guang Wang, Ying Luo, Xian-Qiang Wang, Shao-Cheng Lv, Jia-Hong Dong

**Affiliations:** Hepatobiliary Department, PLA General Hospital of China, Beijing, 100853 China

**Keywords:** Robotic surgery, Laparoscopy, Distal pancreatectomy, Spleen preservation, Minimally invasive surgery

## Abstract

**Background:**

Spleen-preserving laparoscopic distal pancreatectomy is technically challenging. New surgical robotic systems are now available and show promising outcomes but were very recently implemented in China.

**Methods:**

Seven patients underwent laparoscopic distal pancreatectomy using the da Vinci Robotic System (RDP) for benign or borderline malignant pancreatic tumors. Spleen preservation rate, blood loss, and operative complications were assessed.

**Results:**

Mean age was 44.6 ± 13.7 years. Surgery was uneventful in all patients, without conversion to laparotomy. The surgical time (including anesthesia induction, robot docking, operation, and postoperative awaking time) was 460 ± 154 min, while the operation time was 368 ± 126 min. Blood losses were 200 ± 110 mL. The minor (Clavien I+II) complication rate was 14.3 %, and the major (Clavien III+IV) complication rate was 14.3 %, including hemorrhage and pancreatic leakage. The spleen preservation rate was 100 %. All complications were successfully managed and cured. Intraoperative laparoscopic ultrasound examination successfully identified the correct surgical resection margins. Mean postoperative hospitalization was 8.7 ± 6.6 days. No patient had to undergo a second pancreas surgery. Patients were followed up for a median of 6.8 months (range, 6 to 22 months). All patients survived and reported few discomforts.

**Conclusions:**

RDP is feasible and allows the preservation of the splenic vessels.

## Background

Spleen-preserving laparoscopic distal pancreatectomy is currently accepted for the treatment of benign and borderline malignant pancreas tumors located distally. However, preserving splenic vessels during distal pancreatectomy is an important issue. Two procedures (Kimura’s and Warshaw’s) allow spleen preservation during distal pancreatectomy [1, 2]. In the Kimura procedure, the splenic vessels are preserved, ensuring excellent blood supply to the spleen [1]. In the Warshaw procedure, the short gastric and left gastroepiploic arteries and veins are preserved, but the splenic vessels are sacrificed [2]. During traditional laparoscopic distal pancreatectomy (LDP), inherent limits and shortcomings such as limited visibility, poor ergonomics, and limited dexterity may cause a hemorrhage from vessels’ branches, and a conversion to open surgery may occur.

With recent advances in three-dimensional optics and computer-enhanced motion control, robotic-assisted surgery (RAS) achieved the potential to overcome some of the limits observed with LDP. Indeed, it allows performing complex pancreatic resections with improved ergonomics, visualization, precision, and dexterity during spleen-preserving LDP. Previous studies have shown that the use of RAS was as safe and efficient as open surgery [3, 4].

However, robotic distal pancreatectomy (RDP) was implemented only recently in China, and there is a lack of reports about this technique in Chinese patients. In this report, we present our initial RDP experience.

## Methods

Between June 2009 and March 2012, 28 consecutive patients diagnosed with benign or borderline masses in the distal pancreas (by preoperative ultrasound and computer tomography or magnetic resonance imaging) and evaluated by the same group of surgeons at the People’s Liberation Army General Hospital were approached for participation in the present study. After discussion about the pros and cons of RDP vs. LDP, patients chose the approach they wanted, resulting in seven patients choosing RDP Table 1. Patients who underwent LDP were included as controls for comparing some features between RDP and LDP.

Patients underwent robotic-assisted surgery using the da Vinci S system (Intuitive, Sunnyvale, CA, USA).

This study was approved by the Institutional Review Board of the Chinese People’s Liberation Army General Hospital. All possible advantages and disadvantages of RDP were clearly explained to the patients. Written informed consent was obtained from each patient.

### Robotic-assisted distal pancreatectomy

**Fig. 1:**
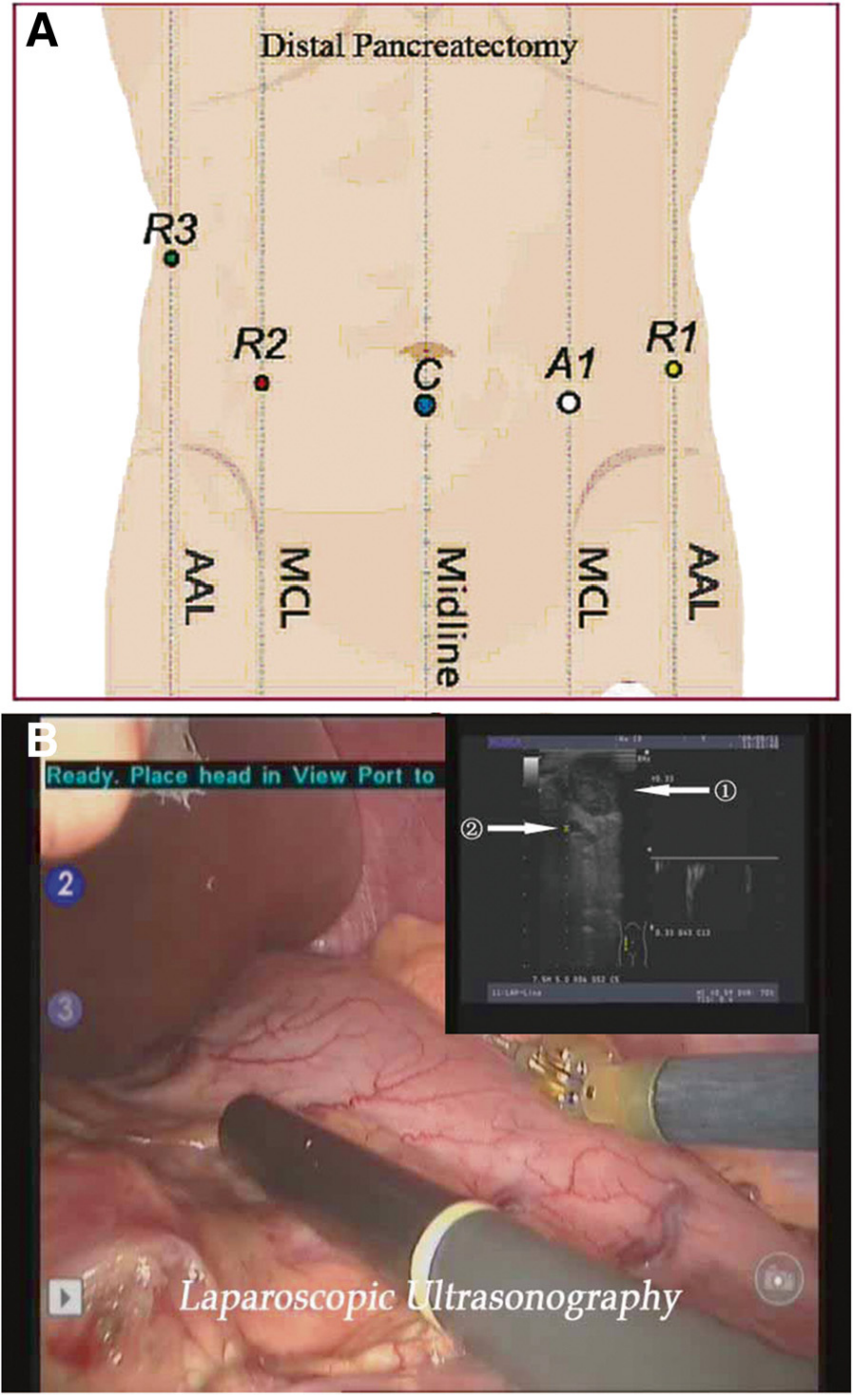
**a** Port placement for RDP. *C* camera port (12 mm), *R1* left robotic instrument port (8 mm), *R2* right robotic instrument port (8 mm), *R3* third robotic instrument port (8 mm), *A1* first assistant port (12 mm), *MCL* midclavicular line, *AAL* anterior axillary line. **b** Intraoperative laparoscopic ultrasound examination of the pancreas. *Arrow 1* shows the tumor. *Arrow 2* shows the splenic vessels

RDP was performed according to the Kimura procedure [1] by a single chief surgeon having about 30 years of experience in pancreatectomy. The patient was positioned in a 30° reverse-Trendelenburg position with his arms tucked at his sides and the legs in the low lithotomy position. An intra-abdominal pressure was established at 14 mmHg using the Veress needle technique. Five operating trocars were placed as shown in Fig. 1a: a 12-mm camera port (C), two 8-mm da Vinci trocars (R2, R3), one 8-mm port (R1), and a 12-mm port (A1) for the assistant.

The lesser sac was entered by dividing the gastrocolic ligament with preservation of the gastroepiploic artery. The pancreas body was then exposed. In order to achieve a better surgical exposure, R3 was used to pull up the stomach, which is a much more stable approach compared with laparoscopy, reducing the need for assistance. After a careful exploration of the peritoneal cavity and viscera, intraoperative laparoscopic ultrasound examination was performed using a diagnostic ultrasound machine (Aloka, Tokyo, Japan) with a UST-5410 variable angle high-frequency linear array probe at 4–13 MHz. The probe was inserted into the abdominal cavity through port R1 to seek for previously undetected lesions and to determine the accurate surgical resection margins (Fig. 1b). Marks were made with an electrotome to define the margins.

**Fig. 2:**
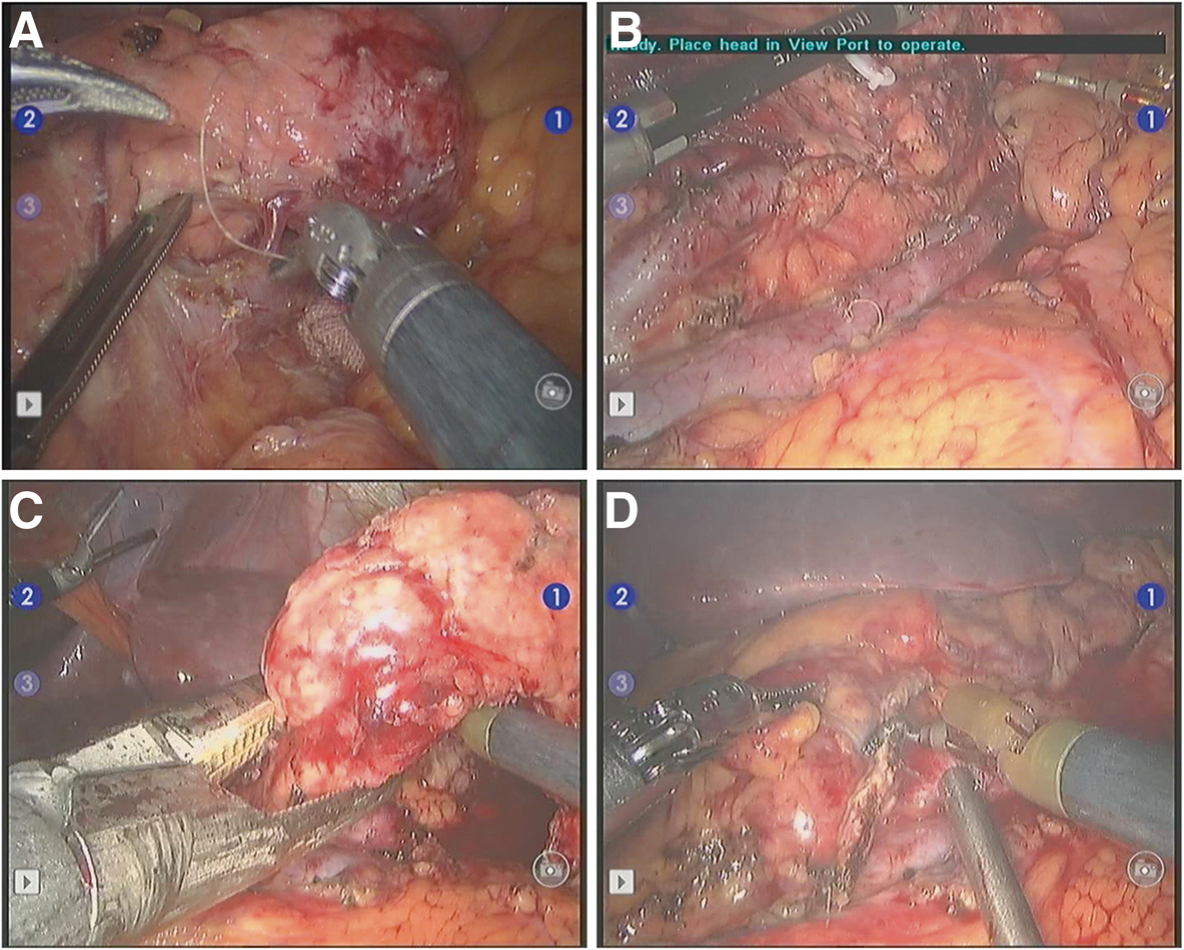
Robotic-assisted spleen-preserving laparoscopic distal pancreatectomy. **a** Ligation of the splenic vein (*arrow*), which passes through the pancreas. **b** The pancreas is totally free from the splenic artery and vein. All branches were treated by sonic shear (<2 mm) or ligature (≥2 mm). Splenic artery and vein were completely preserved. **c** Use of the surgical stapler (EC60) to perform pancreatic dissection (with ≥1-cm margin). The tumor is indicated by an *arrow*. **d** Verification of the pancreatic section, hemorrhage, and pancreatic leak

We isolated the upper and lower edges of the pancreas on the right side of the tumor. The splenic artery and splenic vein branches and tributaries were isolated using an electric coagulation scalpel attached to one of the robotic arms. For vascular control, ligature was more often used, but transfixating sutures were also used if necessary (Fig. 2a). These two hemostatic methods are difficult to perform using LDP but are easy when using RDP [5]. By dividing these structures from the pancreas in a head-to-tail direction, the splenic artery and vein were undamaged, and the blood supply system was completely preserved (Fig. 2b) [1]. An intraoperative ultrasound was performed again to identify the location of the lesion and its relative position with vessels and other organs and to confirm the negative margins. Splenic artery and vein were carefully identified. The dissection then begun from the right side, with margins of 1 cm around the tumor, using a surgical stapler (Echelon 60, Johnson & Johnson, U.S.A, Fig. 2c). The body and tail of the pancreas harboring the tumor were totally dissected. After transection using the Echelon 60 surgical stapler (Johnson & Johnson, USA), three rows of cross pins in each incisional edge were used to tightly block the two transverse pancreatic arteries. This was usually sufficient to control bleeding. If bleeding occurred, 4-0 Prolene suture was used (“8” suture method). Hemostasis was performed on the pancreatic stump by electrocautery or transfixation (Fig. 2d). Due to the learning curve, a slightly improved method for splenic artery and vein identification was used for the later cases: the pancreas body was first transected without any vascular control, and then the splenic vessels were identified and dissected free from the pancreas towards the splenic hilum. Using this approach, splenic artery and vein were easily identified. After irrigation in the surgical bed, and checking for bleeding and pancreatic leak, two drains were systematically placed around the pancreas stump. The specimen was then extracted from the abdomen using a plastic bag.

### Data collection and follow-up

Patients’ demographics, operative time, complications, and length of hospital stay were recorded. The surgical time was calculated as the time between anesthesia and postoperative awaking time (including anesthesia induction, robot docking, operation, and postoperative awaking time). The operative time was calculated as the time between skin incision and the last port skin closure. The exploratory laparoscopy, the robotic set up and docking, and any associated required procedures were included in the surgery time.

Patients were followed up during visits to the outpatient department or by telephone. Follow-up ended at the last visit recorded in the patient’s medical chart. The patients were asked if they felt any discomforts and if their work and daily life were impaired in any manner.

### Statistical analysis

Results are presented as means ± standard deviation. SPSS 15.0 (SPSS Inc., Chicago, IL, USA) was used for statistical analysis. Groups were compared using the Mann-Whitney test or the independent sample *t* test for continuous variables, as appropriate, while the Pearson chi-square test was used for categorical variables. Two-sided *P* values ≤ 0.05 were considered significant.

## Results

The seven patients who underwent RDP (five women and two men; mean age of 44.6 ± 13.7 years, ranging 32–73) suffered from serous cystadenomas (*n* = 4), islet cell tumor (*n* = 1), solid pseudopapilloma (*n* = 1), or mucinous cystadenoma (*n* = 1). The average lesion size was 3.0 ± 0.7 cm (2.5 to 4.0 cm).

All patients underwent the Kimura procedure. In one patient, RDP and a right adrenal tumor resection were performed at the same time. In another patient, RDP and a cholecystectomy were performed at the same time. Therefore, the surgery required more time in these two patients. Intraoperative laparoscopic ultrasound examination successfully and correctly identified surgical resection margins.

All seven RDP procedures were successful. None required a conversion to laparotomy or LPD. Surgical margins were more than 1 cm in all cases. The mean surgical time was 460 ± 154 min (range, 270–720 min). The mean operative time was 368 ± 126 min (range, 220–600 min). Spleen preservation rate was 100 %. Blood losses were 200 ± 110 mL. The mean postoperative stay was 8.7 ± 6.6 days.

Complications were assessed according to the Clavien classification of surgical complications (2004 revised version) [6] and to the International pancreatic fistula research team’s classification of postoperative pancreatic fistula [7]. Two patients (28.6 %) suffered from at least one complication: the minor (Clavien I+II) complication rate was 14.3 %, and the major (Clavien III+IV) complication rate was 14.3 %. Twelve days after surgery, one patient complained of abdominal pain. CT scan showed a 10 × 11 × 11-cm hematoma in the gastric area, above the pancreatic area. Hemoglobin levels were decreased from 127 (preoperative) to 108 g/L. This intra-abdominal hemorrhage was grade II. It was treated using conservative treatment (hemostatics), without blood transfusion or surgery. He was discharged without any subsequent complication. One patient suffered from an intraperitoneal hemorrhage and had to undergo emergency surgery the day after RDP. About 2000 mL of blood, clots, and fluids were removed, but no clear bleeding site could be identified. Bleeding did not recur, and the patient was eventually discharged without any other complications. Four patients suffered from a pancreatic fistula (grade A) detected by amylase values of three times the upper value of normal from the surgical drains. The fistulas did not require percutaneous drainage. Drainage tubes were placed by surgery to drain the fistula. These patients did not receive somatostatin.

Total hospital costs were $10,125 per patient.

Patients were followed up for a median of 6.8 months (range, 6 to 22 months). CT scan with contrast performing 1–2 months after surgery showed no evidence of varicose veins near the hilum of the spleen or gastric fundus nor thrombosis or stenosis. At the end of the follow-up, all patients were alive and had few discomforts.

We also examined the operative characteristics of 21 consecutive patients who declined RDP and underwent LDP. Compared with RDP, mean operative time for LDP was shorter (210 vs. 368 min, *P* = 0.0002), blood losses were comparable (250 vs. 200 mL, *P* = 0.45), complication rates were comparable (33.3 vs. 28.6 %, *P* = 0.82), hospital stay was longer (10.6 vs. 8.7 days, *P* = 0.004), and mean hospital costs were lower ($6921 vs. $10,125, *P* = 0.0002) (Table 2).

**Table 1:** Characteristics of patients who underwent RDP (*n* = 7)

Patient no.	Gender	Age	Diagnosis	Tumor size (cm)	Surgery	Surgical time (min)	Operation time (min)	Bleeding (mL)	Postoperative hospital stay (days)	Complications	Treatment for complications	Follow-up
1	Female	46	Mucinous cystadenoma, (pancreatic cystic tumor)	2 × 2 × 2.5	RDP	720	600	200	22	Bleeding for	Conservative treatment	Survived with few discomforts
										12 days after surgery		
2	Female	44	Pancreatic serous cystadenoma	4 × 2.8 × 2.5	RDP	480	390	20	19	Pancreatic leakage grade A	Drainage. Cured 19 days later	Survived with few discomforts
3	Female	32	Pancreatic serous papillary cystadenoma	3.5 × 3 × 2	RDP	405	330	300	11	Intraperitoneal hemorrhage, pancreatic leakage grade A	Emergency surgery to clean the hematoma	Survived with few discomforts
4	Male	33	1. Pancreatic gland rear solid-pseudopapillary tumor	2.5 × 2 × 2	RDP, cholecystectomy	450	355	300	7	Pancreatic leakage grade A	Drainage. Cured 7 days after being discharged from the hospital with outpatient decannulation	Survived with few discomforts
			2. Chronic cholecystitis with cholesterol polyp									
5	Male	41	Pancreatic serous cystadenoma	2.5 × 2 × 1	RDP	315	255	100	7	Pancreatic leakage grade A	Drainage	Survived with few discomforts
6	Female	73	Right adrenal neoplasms, adenoma sebaceum	2.5 × 2 × 1.5	RDP, right adrenal tumor resection	580	430	200	6	None	–	Survived with few discomforts
7	Female	43	Pancreatic serous cystadenoma (pancreatic gland rear cystic tumor)	5 × 3 × 2.5	RDP	270	220	300	7	None	–	Survived with few discomforts
Mean ± SD	–	44.6 ± 13.7	–	–	–	460 ± 154	369 ± 126	203 ± 110	11.3 ± 6.6	–	–	–

*RDP* robot-assisted distal pancreatectomy, *SD* standard deviation

**Table 2:** Surgical outcomes, complications and hospital costs according to the use of a robot during distal pancreatectomy

	RDP (*n* = 7)	LDP (*n* = 21)	*P* value
Mean operative time (min)	368	210	0.0002
Mean blood loss (mL)	200	250	0.451
Transfusion (no. of patients) (%)	0	0	
Conversion (no. of patients) (%)	0	0	
Reverse operation (no. of patients) (%)	0	1 (4.7)^a^	
Mean postoperative stay (days)	8.7	10.6	0.004
Complications (no. of patients) (%)	2 (28.6)	7 (33.3)	0.815
Grade, Clavien classification	II (1), III (1)	I (5), II (1), III (1)	
Hospital costs ($)	10,125	6921	0.0002

*RDP* robot-assisted distal pancreatectomy, *LDP* laparoscopic distal pancreatectomy

^a^A second operation was required in one patient of the LPD group (4.7 %) who had a postoperation hemorrhage

## Discussion

In the present series of patients treated using RDP at a single institution, surgery was uneventful in all patients, without conversion to laparotomy. The spleen preservation rate was 100 %, the minor (Clavien I+II) complication rate was 57.1 %, and the major (Clavien III+IV) complication rate was 14.3 %. No patient had to undergo a second pancreas surgery. All patients survived and reported few discomforts. This study presents the first Chinese experience using RDP. Intraoperative laparoscopic ultrasound examination was performed to define the surgical resection margins, which could contribute to better outcomes. These results are supported by a previous study by Hwang et al [8]. However, in comparison, the present study showed a slightly shorter operation (369 ± 126 vs. 399 ± 166 min), and blood losses that were nearly halved (203 ± 110 vs. 360 ± 360 mL). However, the hospital stay was longer (11 ± 7 vs. 7 ± 2 days).

Indeed, preserving the immunological functions of the spleen can help to avoid the incidence of leukocytosis, thrombocytosis, overwhelming post-splenectomy sepsis, and low-grade immunodeficiency. A retrospective study from the Memorial Sloan-Kettering Cancer Center in 125 patients who underwent distal pancreatectomy with or without splenectomy showed that there were postoperative complications in 49 % of patients who underwent splenectomy vs. 39 % of patients who did not [9]. In addition, perioperative infectious complications and severe complications were more frequent in the splenectomy group (28 vs. 9 % and 11 vs. 2, respectively). Thus, spleen preservation during distal pancreatectomy for benign or borderline malignant tumors is now an accepted practice.

The Warshaw procedure is one of the two methods for preserving the spleen, but the spleen’s blood supply cannot be guaranteed [10]. In an attempt to alleviate the morbidity associated with splenectomy, we were interested in preserving the whole splenic blood supply during distal pancreatectomy. The Kimura procedure completely preserves the splenic artery and vein, as well as perfusion of the spleen, without noticeable changes in physiological functions. During our follow-up period, we did not observe any evidence of varicose veins near the hilum of the spleen or gastric fundus in RDP patients, which might be better than outcomes achieved using the Warshaw procedure since a previous series reported perigastric varices in 25 % of patients during follow-up after the Warshaw surgery [10]. However, this procedure is difficult to perform using traditional laparoscopy, and it is much easier to accomplish using a robotic laparoscopy approach [5].

Since the technology allowing this approach is relatively novel, RDP is believed to be an uncommon surgical procedure. Since Olah et al. [11] first reported “robotic resection of a pancreatic neuroendocrine tumor” in 2003, Giulianotti et al. [12] reported five pancreatic resections using RDP with an average surgical time of 270 min (range, 210–360 min). Among these five cases, only two patients (one for an insulinoma and one for a benign cystic lesion) underwent spleen-preserving pancreatic resection. Some other studies are also available from different countries comparing RDP vs. LDP vs. open surgery, with or without splenic vessel preservation [13–17]. A number of case reports and small series are also available.

Robotic surgery offers the opportunity to combine the advantages of both minimally invasive and open surgical approaches [4]. The patients promptly return to full activity and have a short hospital stay. For the surgeons, RDP has the advantage of requiring less laparoscopic experience. Furthermore, the dissection of the splenic vein and artery and the creation of the retropancreatic tunnel were more easily performed using the da Vinci system compared with LDP [18]. Tumors in the body and tail of the pancreas are commonly treated with minimally invasive surgical techniques, and the advantage of decreased tissue trauma may prove to be beneficial for a patient with a benign disease. Thus, a minimally invasive approach should be advocated for this type of disease, particularly because of the benefits it provides in terms of postoperative outcomes, such as improved respiratory function and less operative stress.

According to our experience, the key feature during RDP is freeing the upper and lower edges of the pancreas followed by venous ligation or transfixion of the vessels close to the hilus of the spleen. Intra-operative venous bleeding can usually be managed with a combination of direct electric coagulation or sutures. Theoretically, using a robot can greatly increase the ability to deal with small and delicate branches of splenic vessels because of the superior visualization and precision offered by the da Vinci surgical system. Nevertheless, postoperative hemorrhage may occur, and hemorrhage is the most common and serious complication of this surgery.

We used the EC60 stapler for managing the pancreatic stump because this method is simple, quick, and known to be a safe alternative to the standard suture closure technique [19]. We usually use the gold cartridge to cutoff the pancreas because the staple length is 3.8 mm and the closed height is then 1.8 mm, making the pancreatic stump wrinkled, reducing the incidence of pancreatic fistula.

However, using a robot presents a number of disadvantages. The surgeons who control the robot in the surgical console do not feel any touch sensation associated with physically touching the tissues, and they have to rely on visual feedback only [20]. It is common to encounter a hemorrhage while grasping or pulling the pancreas during the division of small splenic vessels from the pancreas. Sutures or electro-coagulation using Biclamp are two good options for hemostasis, but these procedures are onerous in terms of surgical time. However, compared with laparoscopy, suture of the spleen is not so risky anymore [5]. The setup of the robotic arms necessitates good spatial planning and is time-consuming, especially at the beginning of the learning curve [13]. Pancreatic fistula is one of the complications that may be encountered during RDP, but it is usually manageable by drainage. For overweight patients, setup and pancreatic exposure may not be easy, and one or two supplementary ports might be necessary. Another important criticism is the higher cost of RDP compared with LDP and is one of the main obstacles for the use of RDP in usual clinical practice [5, 13].

In the present study, 71 % (5/7) of the patients suffered from complications. A previous study reported a complication rate of 26 % after robotic pancreatic surgery [4], but it was reported after performing robotic pancreatectomy in 134 patients (compared with 7 in the present study), which may suggest an effect of the learning curve on the incidence of complications. In addition, this previous study [4] included all types of pancreatectomy, while the present study focused on RDP with preservation of the splenic vessels. A previous study in 246 patients who underwent laparoscopic distal pancreatectomy showed a complication rate of 31.3 % [21]. Previous studies also reported similar complication rates between RDP and LDP [17, 22]. It might be expected that with more experience, the complication rate of RDP should decrease.

RDP has been implemented in some rare centers in China only a few years ago. With experience and improvements of this technique, it is anticipated that the hospital stay, complication rate, and costs should be improved, making it more attractive than traditional approaches. Nevertheless, in the context of the Chinese healthcare system, the hospital stay was not overtly long. In addition, if the first two patients were excluded, the mean hospital stay would be significantly shorter, and the complication rates would be lower. Seemingly, in this study and in contrast to LDP, RDP could improve some operative characteristics (e.g., blood losses) but increased the mean operative time, complication rate, and mean hospital costs. However, since the characteristics of the patients (e.g., age, disease type, and lesion size) were not comparable between the RDP and LDP groups, the treatment efficacy between the two procedures could not be compared directly.

The present study has some limitations. Even if we compared two groups of patients, the sample size was too small to draw any firm conclusion. Since the patients chose which surgical approach they wanted, based on safety and costs, it is possible that the patients choosing the robotic approach had a higher income than those choosing LDP, as well as a standard of living favoring good outcomes. However, the present report is a case series presenting our initial experience in a newly implemented technology in China. We are currently conducting a prospective trial in a larger number of patients. In the present study, postoperative stays were particularly long. However, this issue lays in the Chinese healthcare system, which requires that all patients are completely symptom-free upon discharge, which mean without any abdominal cavity drainage tube. In addition, even mild discomfort expressed by the patient will make the hospital unwilling to discharge him.

## Conclusions

Our preliminary real-life study suggests that RDP is a feasible and lowly invasive option for the resection of benign or borderline malignant tumors of the pancreas. We used the Kimura procedure with RDP, and results suggest that it is beneficial for preserving the splenic vessels. However, postoperative complication rate and costs were high, and hospital stay and surgical time were long. Additional multiple-center randomized prospective studies with a larger number of patients are required to assess the efficacy of RDP.

## Abbreviations

LDP: laparoscopic distal pancreatectomy
RAS: robotic-assisted surgery
RDP: robotic distal pancreatectomy
